# Supplementary material for: High-density cortical µECoG arrays concurrently track spreading depolarizations and long-term evolution of stroke in awake rats
Source: Commun Biol. 2024 Mar 4;7:263. doi: 10.1038/s42003-024-05932-0 (PMC10912118; doi:10.1038/s42003-024-05932-0)
Supplement: Supplementary file 2 — Supplementary Information [file 42003_2024_5932_MOESM2_ESM.pdf]

High-density cortical µECoG arrays concurrently track spreading depolarizations and long-term evolution of stroke in awake rats

Supplementary Information

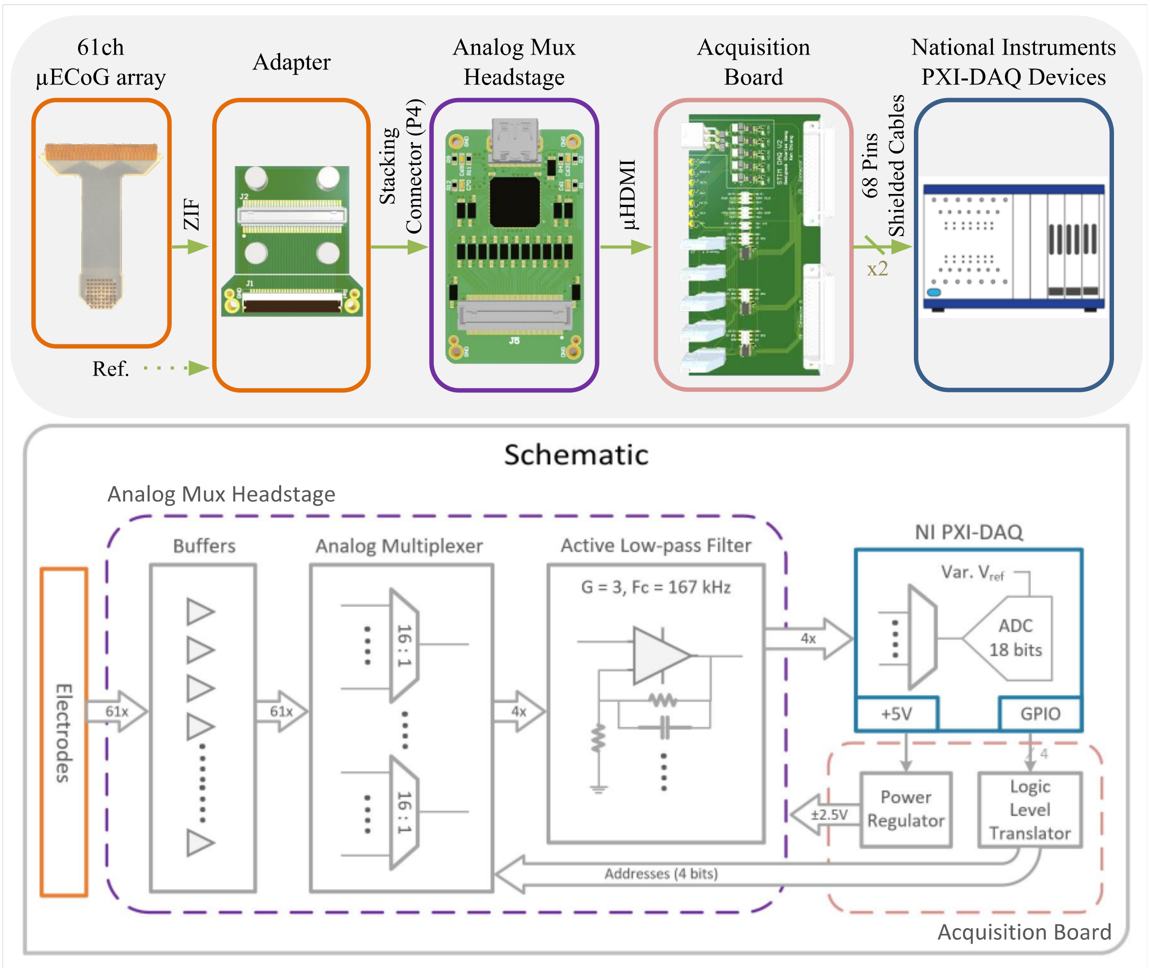

Supplementary Figure 1 | Overview of recording hardware and accompanying schematic. Recordings are DC-coupled.

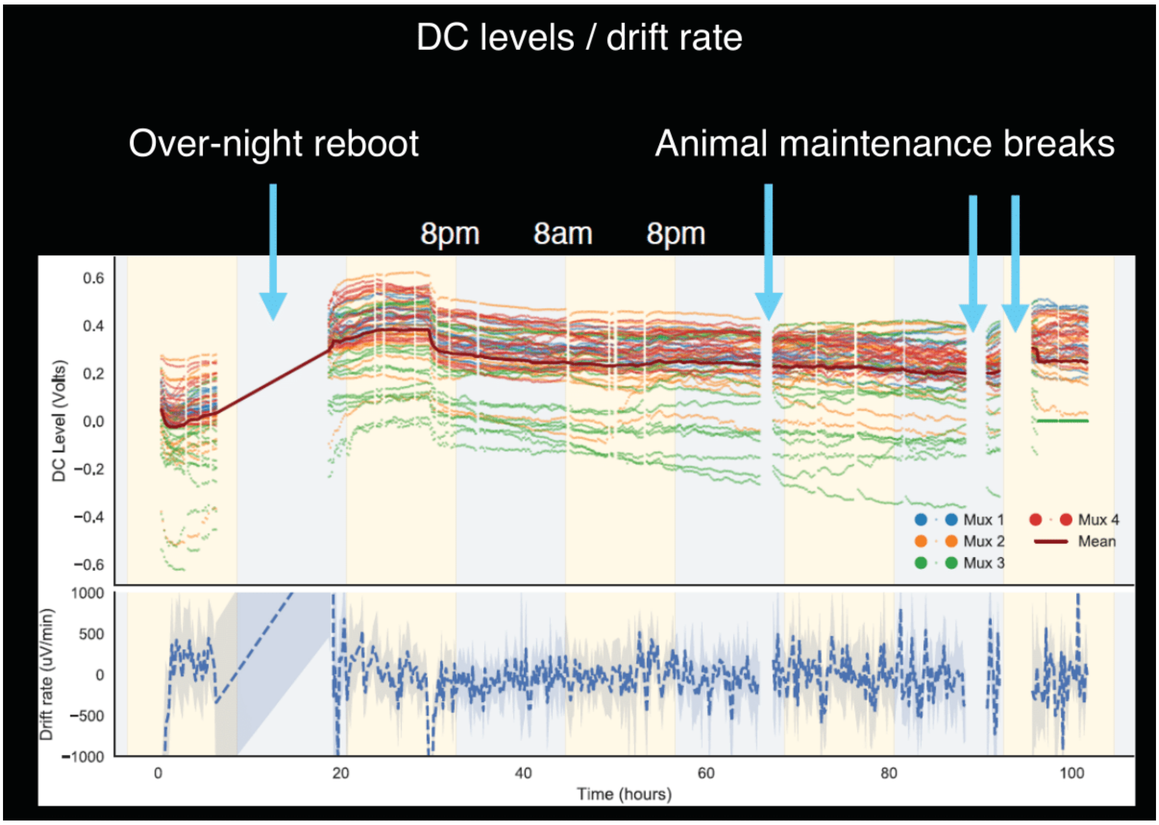

Supplementary Figure 2 | DC drift rate of recording system, as recorded from a fully implanted and freely behaving animal.

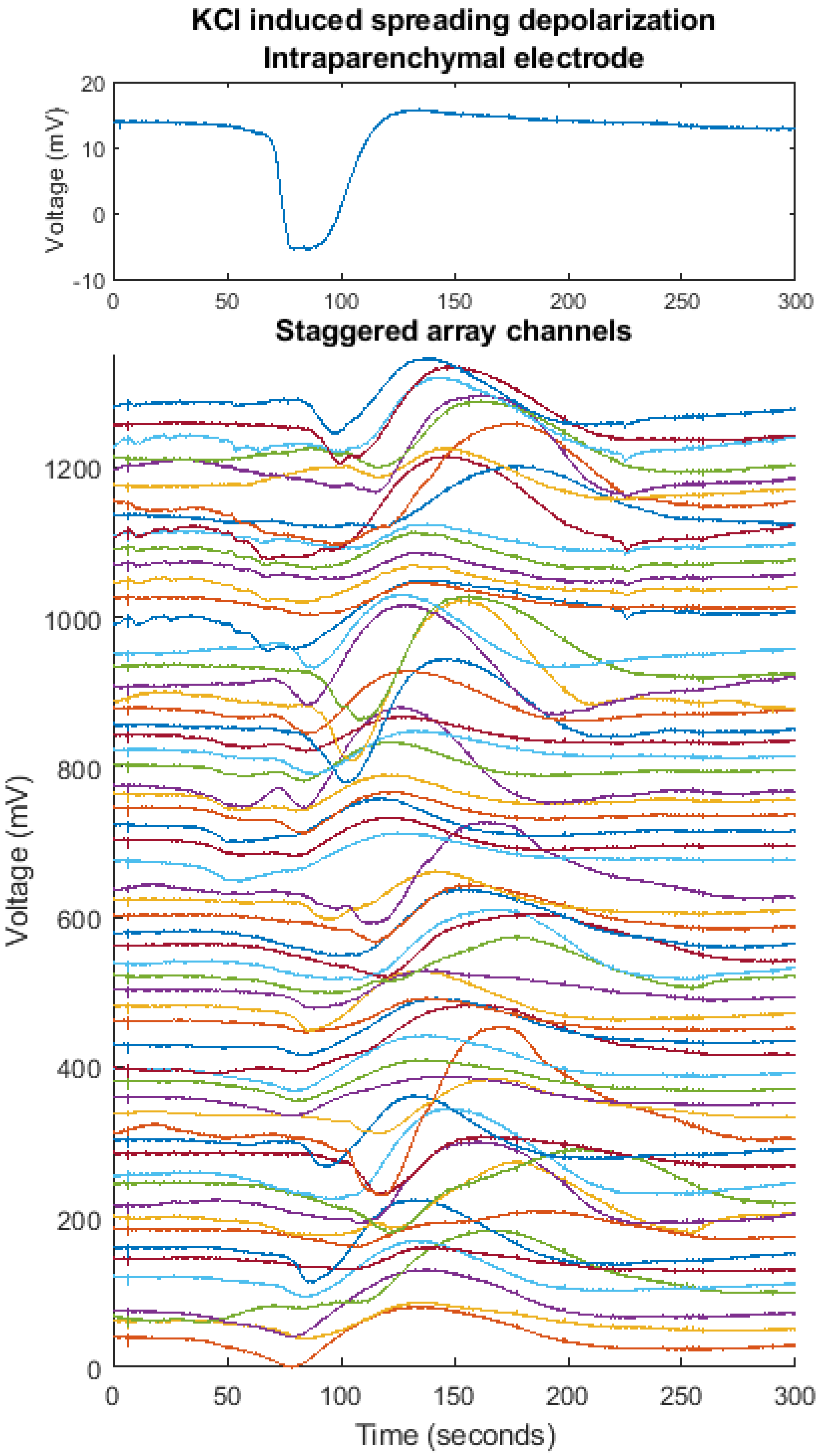

Supplementary Figure 3 | KCl-induced SD, recorded simultaneously with an intraparenchymal electrode and a µECoG array.

Supplementary Notes 1:

Statistical analysis for main text Figure 2 (conducted in JMP Pro 15)

Global ANOVA

Factors

1. Condition (3 levels: baseline, stroke, post-mortem)
2. Frequency (3 levels: low (<0.5 Hz), middle (0.5-20 Hz), high (>20 Hz)

Effect tests

Source	Nparm	DF	Sum of Squares	F Ratio	Prob > F
Condition	2	2	18.618872	269.0715	3.9e-14
Frequency	2	2	25.812081	373.0245	2.24e-15
Condition*Frequency	4	4	6.247911	45.1460	3.793e-9

Follow-up ANOVAs (subdividing the data by frequency)

Factors

1. Condition (3 levels: baseline, stroke, post-mortem)

Low (<0.5 Hz) frequency

Effect tests

Source	Nparm	DF	Sum of Squares	F Ratio	Prob > F
Condition	2	2	10.911688	55.4795	0.000135

Post-hoc test Tukey HSD

Level				Least Sq Mean
Stroke	A			-0.360636
Baseline		B		-2.009185
Post-mortem			C	-3.033574

Levels not connected by same letter are significantly different.

Level	- Level	Difference	Std Err Dif	Lower CL	Upper CL	p-Value
Stroke	Post-mortem	2.672938	0.2560466	1.887349	3.458528	0.000112
Stroke	Baseline	1.648549	0.2560466	0.862960	2.434138	0.001615
Baseline	Post-mortem	1.024389	0.2560466	0.238800	1.809978	0.016723

Middle (0.5-20 Hz) frequency

Effect tests

Source	Nparm	DF	Sum of Squares	F Ratio	Prob > F
Condition	2	2	12.906384	1510.155	7.793e-9

Post-hoc test Tukey HSD

Level				Least Sq Mean
Baseline	A			-2.608307
Stroke		B		-2.802831
Post-mortem			C	-5.240290

Levels not connected by same letter are significantly different.

Level	- Level	Difference	Std Err Dif	Lower CL	Upper CL	p-Value
Baseline	Post-mortem	2.631983	0.0533741	2.468223	2.795743	1.17e-8
Stroke	Post-mortem	2.437460	0.0533741	2.273700	2.601219	1.847e-8
Baseline	Stroke	0.194523	0.0533741	0.030764	0.358283	0.025078

High (>20 Hz) frequency

Effect tests

Source	Nparm	DF	Sum of Squares	F Ratio	Prob > F
Condition	2	2	1.0487121	443.5626	3.032e-7

Post-hoc test Tukey HSD

Level				Least Sq Mean
Stroke	A			-3.740036
Baseline		B		-3.982909
Post-mortem			C	-4.554376

Levels not connected by same letter are significantly different.

Level	- Level	Difference	Std Err Dif	Lower CL	Upper CL	p-Value
Stroke	Post-mortem	0.8143402	0.0280731	0.7282078	0.9004726	2.765e-7
Baseline	Post-mortem	0.5714675	0.0280731	0.4853351	0.6575999	2.268e-6
Stroke	Baseline	0.2428727	0.0280731	0.1567403	0.3290051	0.000323

Supplementary Notes 2:

Statistical analysis for main text Figure 3 (conducted in JMP Pro 15)

ANOVA

Factors

1. Condition (5 levels: baseline, 1% isoflurane, 2% isoflurane, 3% isoflurane, post-mortem)

Effect tests

Source	Nparm	DF	Sum of Squares	F Ratio	Prob > F
Condition	4	4	21.494272	1138.446	3.03e-13

Post-hoc test Tukey HSD

Level					Least Sq Mean
iso1	A				-8.36564
iso2		B			-9.38567
baseline		B			-9.51830
iso3			C		-10.65357
post-mortem				D	-11.86179

Levels not connected by same letter are significantly different.

Level	- Level	Difference	Std Err Dif	Lower CL	Upper CL	p-Value
iso1	post-mortem	3.496154	0.0560957	3.31154	3.680770	1.44e-11
iso2	post-mortem	2.476124	0.0560957	2.29151	2.660740	2.09e-11
baseline	post-mortem	2.343497	0.0560957	2.15888	2.528112	2.59e-11
iso1	iso3	2.287931	0.0560957	2.10332	2.472547	2.91e-11
iso2	iso3	1.267901	0.0560957	1.08329	1.452517	5.142e-9
iso3	post-mortem	1.208223	0.0560957	1.02361	1.392839	8.24e-9
iso1	baseline	1.152658	0.0560957	0.96804	1.337273	1.305e-8
baseline	iso3	1.135274	0.0560957	0.95066	1.319889	1.514e-8
iso1	iso2	1.020030	0.0560957	0.83541	1.204646	4.292e-8
iso2	baseline	0.132627	0.0560957	-0.05199	0.317243	0.202357

Supplementary Notes 3:

Statistical analysis for main text Figures 5 and 7 (conducted in MATLAB)

Cluster-based nonparametric tests were conducted using the MATLAB function “permutest.”

> Edden M. Gerber (2023).
> permutest (https://www.mathworks.com/matlabcentral/fileexchange/71737-permutest),
> MATLAB Central File Exchange. Retrieved October 6, 2023.

This function was based on the publication by Maris and Oostenveld (2007).

> Maris, E., & Oostenveld, R. (2007). Nonparametric statistical testing of EEG-and MEG-data.
> Journal of Neuroscience Methods, 164(1), 177–190.
>
> https://doi.org/10.1016/j.jneumeth.2007.03.024

We used the following inputs for “permutest:”

- dependent_samples = true
- p_threshold = 0.05
- num_permutations = 1000
- two_sided = true
- num_clusters = inf

Supplementary Videos 1-3 (that accompany Figures 4 and 6) are uploaded separately.

Supplementary Notes 4:

Statistical analysis for main text Figure 8 (conducted in JMP Pro 15)

Global ANOVA

Factors

1. Time (3 levels: panel I, panel IV pre-mortem, panel IV post-mortem)
2. Channel (3 levels: 1, 2 3)
3. Frequency (3 levels: low (<0.5 Hz), middle (0.5-20 Hz), high (>20 Hz)

Effect tests

Source	Nparm	DF	Sum of Squares	F Ratio	Prob > F
Time	2	2	48.33295	207.9469	5.9627e-42
Channel	2	2	275.90953	1187.069	2.1361e-86
Time*Channel	4	4	24.18099	52.0180	1.9127e-26
Frequency	2	2	26.24196	112.9030	1.519e-29
Time*Frequency	4	4	6.17710	13.2881	3.72059e-9
Channel*Frequency	4	4	85.33314	183.5680	1.4726e-53
Time*Channel*Frequency	8	8	4.00862	4.3117	0.00012006

Follow-up ANOVAs (subdividing the data by channel)

Factors

1. Time (3 levels: panel I, panel IV pre-mortem, panel IV post-mortem)
2. Frequency (3 levels: low (<0.5 Hz), middle (0.5-20 Hz), high (>20 Hz)

Channel 1

Effect tests

Source	Nparm	DF	Sum of Squares	F Ratio	Prob > F
Time	2	2	44.257915	106.8943	8.05e-18
Frequency	2	2	68.151157	164.6027	2.01e-21
Time*Frequency	4	4	6.109406	7.3779	0.000117

Post-hoc test Tukey HSD

Level				Least Sq Mean
'TimePoint1'	A			-7.241616
'TimePoint2'		B		-8.087842
'TimePoint3'			C	-9.439859

Levels not connected by same letter are significantly different.

Level	- Level	Difference	Std Err Dif	Lower CL	Upper CL	p-Value
'TimePoint1'	'TimePoint3'	2.198243	0.1516638	1.830668	2.565817	1.2e-11
'TimePoint2'	'TimePoint3'	1.352017	0.1516638	0.984442	1.719591	6.21e-11
'TimePoint1'	'TimePoint2'	0.846226	0.1516638	0.478652	1.213801	3.871e-6

Channel 2

Effect tests

Source	Nparm	DF	Sum of Squares	F Ratio	Prob > F
Time	2	2	28.243266	103.5801	1.44e-17
Frequency	2	2	21.747196	79.7563	1.61e-15
Time*Frequency	4	4	3.955385	7.2530	0.000135

Post-hoc test Tukey HSD

Level				Least Sq Mean
'TimePoint1'	A			-8.038513
'TimePoint2'		B		-8.500322
'TimePoint3'			C	-9.750516

Levels not connected by same letter are significantly different.

Level	- Level	Difference	Std Err Dif	Lower CL	Upper CL	p-Value
'TimePoint1'	'TimePoint3'	1.712004	0.1230787	1.413708	2.010299	1.2e-11
'TimePoint2'	'TimePoint3'	1.250195	0.1230787	0.951899	1.548490	1.3e-11
'TimePoint1'	'TimePoint2'	0.461809	0.1230787	0.163514	0.760105	0.001425

Channel 3

Effect tests

Source	Nparm	DF	Sum of Squares	F Ratio	Prob > F
Time	2	2	0.012762	1.2059	0.308906
Frequency	2	2	21.676747	2048.295	6.47e-45
Time*Frequency	4	4	0.120937	5.7138	0.000832

Post-hoc test Tukey HSD

Level		Least Sq Mean
'TimePoint1'	A	-11.22189
'TimePoint2'	A	-11.25013
'TimePoint3'	A	-11.25759

Levels not connected by same letter are significantly different.

Level	- Level	Difference	Std Err Dif	Lower CL	Upper CL	p-Value
'TimePoint1'	'TimePoint3'	0.0356961	0.0242474	-0.023070	0.0944623	0.313732
'TimePoint1'	'TimePoint2'	0.0282332	0.0242474	-0.030533	0.0869995	0.480333
'TimePoint2'	'TimePoint3'	0.0074629	0.0242474	-0.051303	0.0662292	0.949181
