# Supplementary material for: High-density cortical µECoG arrays concurrently track spreading depolarizations and long-term evolution of stroke in awake rats
Source: Commun Biol. 2024 Mar 4;7:263. doi: 10.1038/s42003-024-05932-0 (PMC10912118; doi:10.1038/s42003-024-05932-0)
Supplement: Supplementary file 3 — Description of Additional Supplementary Files [file 42003_2024_5932_MOESM3_ESM.pdf]

Description of Additional Supplementary Files

File name: Supplementary Video 1
Description: Movie for Figure 4.

File name: Supplementary Video 2
Description: Movie for Figure 6b.

File name: Supplementary Video 3
Description: Movie for Figure 6c.
