# Supplementary material for: High-density cortical µECoG arrays concurrently track spreading depolarizations and long-term evolution of stroke in awake rats
Source: Commun Biol. 2024 Mar 4;7:263. doi: 10.1038/s42003-024-05932-0 (PMC10912118; doi:10.1038/s42003-024-05932-0)
Supplement: Supplementary file 7 — Reporting Summary [file 42003_2024_5932_MOESM7_ESM.pdf]

nature portfolio

Corresponding author(s): Ulrike Hoffmann

Last updated by author(s): Dec 28, 2023

Reporting Summary

Nature Portfolio wishes to improve the reproducibility of the work that we publish. This form provides structure for consistency and transparency in reporting. For further information on Nature Portfolio policies, see our Editorial Policies and the Editorial Policy Checklist.

Statistics

For all statistical analyses, confirm that the following items are present in the figure legend, table legend, main text, or Methods section.

n/a	Confirmed	
☐	☒	The exact sample size (*n*) for each experimental group/condition, given as a discrete number and unit of measurement
☐	☒	A statement on whether measurements were taken from distinct samples or whether the same sample was measured repeatedly
☒	☐	The statistical test(s) used AND whether they are one- or two-sided<br>*Only common tests should be described solely by name; describe more complex techniques in the Methods section.*
☒	☐	A description of all covariates tested
☒	☐	A description of any assumptions or corrections, such as tests of normality and adjustment for multiple comparisons
☐	☒	A full description of the statistical parameters including central tendency (e.g. means) or other basic estimates (e.g. regression coefficient) AND variation (e.g. standard deviation) or associated estimates of uncertainty (e.g. confidence intervals)
☒	☐	For null hypothesis testing, the test statistic (e.g. *F*, *t*, *r*) with confidence intervals, effect sizes, degrees of freedom and *P* value noted<br>*Give P values as exact values whenever suitable.*
☒	☐	For Bayesian analysis, information on the choice of priors and Markov chain Monte Carlo settings
☒	☐	For hierarchical and complex designs, identification of the appropriate level for tests and full reporting of outcomes
☒	☐	Estimates of effect sizes (e.g. Cohen's *d*, Pearson's *r*), indicating how they were calculated

Our web collection on statistics for biologists contains articles on many of the points above.

Software and code

Policy information about availability of computer code

Data collection	A custom data acquisition system and software were used to collect the data. Both of these have been previously published, and the software has an open source license.<br><br>H. Bink, J. B. Wagenaar and J. Viventi, "Data acquisition system for high resolution, multiplexed electrode arrays," 2013 6th International IEEE/EMBS Conference on Neural Engineering (NER), San Diego, CA, USA, 2013, pp. 1001-1004, doi: 10.1109/NER.2013.6696105.<br><br>Insanally, M., et al., A low-cost, multiplexed muECoG system for high-density recordings in freely 843 moving rodents. J Neural Eng, 2016. 13(2): p. 026030-26030. DOI 10.1088/1741-2560/13/2/026030
Data analysis	All data analysis was completed in MATLAB or Python with standard packages.

For manuscripts utilizing custom algorithms or software that are central to the research but not yet described in published literature, software must be made available to editors and reviewers. We strongly encourage code deposition in a community repository (e.g. GitHub). See the Nature Portfolio guidelines for submitting code & software for further information.

Data

Policy information about availability of data

All manuscripts must include a data availability statement. This statement should provide the following information, where applicable:

- Accession codes, unique identifiers, or web links for publicly available datasets
- A description of any restrictions on data availability
- For clinical datasets or third party data, please ensure that the statement adheres to our policy

Data are available at request from the corresponding authors.

Research involving human participants, their data, or biological material

Policy information about studies with human participants or human data. See also policy information about sex, gender (identity/presentation), and sexual orientation and race, ethnicity and racism.

Reporting on sex and gender	N/A
Reporting on race, ethnicity, or other socially relevant groupings	N/A
Population characteristics	N/A
Recruitment	N/A
Ethics oversight	N/A

Note that full information on the approval of the study protocol must also be provided in the manuscript.

Field-specific reporting

Please select the one below that is the best fit for your research. If you are not sure, read the appropriate sections before making your selection.

☒ Life sciences ☐ Behavioural & social sciences ☐ Ecological, evolutionary & environmental sciences

For a reference copy of the document with all sections, see nature.com/documents/nr-reporting-summary-flat.pdf

Life sciences study design

All studies must disclose on these points even when the disclosure is negative.

Sample size	No sample-size calculation was performed. The purpose of the study was to develop and validate new instrumentation and technology, and not to compare outcomes between multiple experimental groups. Technical pilot experiments (n=7) were performed until the novel instrumentation was finalized. All subsequent experiments (n=13) confirmed that the novel instrumentation worked as intended, and after 13 successful replications of these findings, the study ended.
Data exclusions	echnical pilot experiments were reported in the manuscript, but excluded from data analysis. The reason for exclusion is that the technical pilot experiments were used for fine-tuning and adjustment of the novel instrumentation and technology described in the manuscript. Once the novel instrumentation was finalized, all subsequent experiments were included in the data analysis.
Replication	After technical pilot experiments were completed and the novel instrumentation was finalized, all attempts at replication were successful (i.e. our novel instrumentation was reliable and successful in long-term recordings of post-stroke brain signals).
Randomization	Since the purpose of this study was to develop and validate new instrumentation and technology, randomization and experimental groups were not relevant to this study. All animals received a stroke and subsequent monitoring of brain signals.
Blinding	Investigators were not blinded. Blinding was not relevant to this study because all animals received a stroke and subsequent monitoring of brain signals. The purpose of the study was to develop and validate new instrumentation and technology, and not to compare outcomes between multiple experimental groups.

Reporting for specific materials, systems and methods

We require information from authors about some types of materials, experimental systems and methods used in many studies. Here, indicate whether each material, system or method listed is relevant to your study. If you are not sure if a list item applies to your research, read the appropriate section before selecting a response.

Materials & experimental systems

n/a	Involved in the study
☒	☐ Antibodies
☒	☐ Eukaryotic cell lines
☒	☐ Palaeontology and archaeology
☐	☒ Animals and other organisms
☒	☐ Clinical data
☒	☐ Dual use research of concern
☒	☐ Plants

Methods

n/a	Involved in the study
☒	☐ ChIP-seq
☒	☐ Flow cytometry
☒	☐ MRI-based neuroimaging

Animals and other research organisms

Policy information about studies involving animals; ARRIVE guidelines recommended for reporting animal research, and Sex and Gender in Research

Laboratory animals	Wistar rats, age 10-12 weeks.
Wild animals	This study did not involve wild animals.
Reporting on sex	Only male rats were included in this study. Sex-based analysis was not necessary in this study, since the purpose of the study was to develop and validate new instrumentation and technology.
Field-collected samples	This study did not involve samples collected from the field.
Ethics oversight	This study was approved by the Duke University Animal Care and Use Committee.

Note that full information on the approval of the study protocol must also be provided in the manuscript.

Plants

Seed stocks	N/A
Novel plant genotypes	N/A
Authentication	N/A
